# Short-chain fatty acids directly exert anti-inflammatory responses in podocytes and tubular epithelial cells exposed to high glucose

**DOI:** 10.3389/fcell.2023.1182570

**Published:** 2023-05-04

**Authors:** Yan Jun Li, Jin Ma, Yik Wen Loh, Steven J. Chadban, Huiling Wu

**Affiliations:** ^1^ Kidney Node Laboratory, The Charles Perkins Centre, University of Sydney, Darlington, NSW, Australia; ^2^ Sydney Medical School, Faculty of Medicine and Health, University of Sydney, Sydney, NSW, Australia; ^3^ Renal Medicine, Royal Prince Alfred Hospital, Sydney, NSW, Australia

**Keywords:** diabetic kidney disease, gut microbiome, inflammation, short-chain fatty acids, signalling

## Abstract

**Aims:** Gut-microbiome derived short-chain fatty acids exert anti-inflammatory effects and delay progression of kidney disease in diabetic nephropathy. The aim of this study was to examine the impact *in vivo* and *in vitro* of short-chain fatty acid treatment on cellular pathways involved in the development of experimental diabetic nephropathy.

**Methods:** To determine the effect of short-chain fatty acids in diabetic nephropathy, we compared wildtype, GPR43−/− and GPR109A−/− mice diabetic mice treated with acetate or butyrate and assessed variables of kidney damage. We also examined the impact of short-chain fatty acid treatment on gene expression in renal tubular cells and podocytes under high glucose conditions.

**Results:** Short-chain fatty acid treatment with acetate or butyrate protected wild-type mice against development of diabetic nephropathy, exhibiting less glomerular hypertrophy, hypercellularity and interstitial fibrosis compared to diabetic controls. Acetate and butyrate treatment did not provide the same degree of protection in diabetic GPR43−/− and GPR109A−/− diabetic mice respectively. Consistent with our *in vivo* results, expression of pro-inflammatory genes in tubular epithelial cells exposed to high glucose were attenuated by acetate and butyrate treatment. Acetate did not reduce inflammatory or fibrotic responses in glucose stimulated GPR43−/− TECs. Butyrate mediated inhibition of pro-fibrotic gene expression in TECs through GPR109A, and in podocytes via GPR43.

**Conclusion:** SCFAs protect against progression of diabetic nephropathy and diminish podocyte and tubular epithelial injury and interstitial fibrosis via direct, GPR-pathway dependent effects on intrinsic kidney cells. GPR43 and GPR109A are critical to short-chain fatty acid mediated reno-protection and have potential to be harnessed as a therapeutic target in diabetic nephropathy.

## Introduction

Diabetic nephropathy (DN) is the leading cause of chronic kidney disease (CKD) worldwide and continues to grow in incidence and prevalence (Cho et al., 2018). Epidemiological studies support a link between dietary fibre intake and systemic inflammation and mortality in CKD (Krishnamurthy et al., 2012). Gut-derived metabolites from microbial fermentation of dietary fibre such as short-chain fatty acids (SCFA) have shown promise as an inflammatory modulator in autoimmune and inflammatory disease (Lynch and Pedersen, 2016). Treatment with SCFAs in pre-clinical models of AKI and CKD have shown a capacity to prevent or attenuate kidney injury (Andrade-Oliveira et al., 2015; Li et al., 2020; Liu et al., 2021). SCFAs are known to modulate innate and adaptive immune responses both locally within the gastro-intestinal tract and at distant sites (Smith et al., 2013; Koh et al., 2016). However, identification and localization of specific pathways through which SCFAs exert their reno-protective effects remain incompletely characterized.

SCFAs modulate biological host responses by interacting with specific G protein-coupled receptors (GPR) or as histone deacetylase (HDAC) inhibitors (Tan et al., 2014). The major GPRs known to be activated by SCFAs are GPR43, GPR41 and GPR109A (Pluznick et al., 2013; Kobayashi et al., 2017; Felizardo et al., 2019). GPR109A is expressed on podocytes and participates in butyrate mediated reno-protective signaling pathways in Adriamycin nephropathy (Felizardo et al., 2019). In mesangial cells, exposure to the SCFA acetate or a GPR43 agonist inhibited oxidative stress and inflammatory responses under high glucose conditions (Huang et al., 2017). We have previously demonstrated that SCFAs afford protection against progression of diabetic nephropathy in a murine model of T1DM (Li et al., 2020). Whether SCFAs mediated such protection through local (gut) or distant (intrinsic cells, immune cells) targets is not known.

Here we investigated the cellular signaling pathways of SCFAs utilizing *in vivo* and *in vitro* models of diabetic nephropathy. Our findings reveal novel mechanisms of action at the level of intrinsic kidney cells and highlight the therapeutic promise of non-pharmaceutical approaches utilising diet and bacterial metabolites, in the treatment of DN.

## Materials and methods

### Animals

Male Wild-type (WT) C57BL/6 mice were obtained from the Animal Resource Centre (Perth, Western Australia). *Gpr43* ^−/−^ and *Gpr109A* ^−/−^ mice on a C57BL/6 background were bred and maintained in our facility. Animals were housed in a specific pathogen-free facility at the University of Sydney. Mice aged 7–9 weeks old were used. All experiments were performed in accordance with approval from the University of Sydney Animal Ethics Committee. Animals from *in vivo* experiments are from a sample examined previously, however with additional unpublished histological assessment (Li et al., 2020).

### Induction of diabetes

Mice were fasted for 4 h prior to intraperitoneal injection of Streptozotocin (STZ), 55 mg/kg daily, for 5 consecutive days. Non-diabetic controls received volume and pH matched citrate buffer. Mice with a blood glucose level (BGL) > 20 mmol/L were used to assess diabetic kidney injury. Blood glucose was measured with an Accu-Check glucometer (Roche Diagnostics) using tail vein blood. All mice were euthanized 12 weeks after injection.

### SCFA treatment

Three weeks after STZ injection, mice were randomized to receive sodium acetate (SA) 100mM, sodium butyrate (SB) 50 mM or sodium propionate (SP) 100 mM (Sigma-Aldrich) *ad libitum* in drinking water. Control mice received pH and sodium matched water. Drinking solutions were refreshed three times per week.

### Histology

Periodic acid-Schiff’s (PAS) and Picro-Sirius Red (PSR) staining were performed on 3 μm and 5 μm formalin-fixed kidney sections respectively. Glomerular tuft area (A_G_) was measured in 20 glomerular profiles per mouse using DP2-BSW v2.2 (OLYMPUS). Glomerular volume (V_G_) was calculated using the formula: VG = (β/κ) × (AG) ^3/2^, where *β* = 1.38 (shape coefficient for spheres) and *κ* = 1.1 (size distribution coefficient) (Weibel and Gomez, 1962). Glomerular extracellular matrix (ECM) was defined as the PAS-positive area quantified by image analysis software (ImagePro Premier 9). Interstitial collagen was assessed by point counting using an ocular grid at ×400 magnification in 20 consecutive fields of renal cortex. Fibrosis score was expressed as percentage of positive crosses (McWhinnie et al., 1986).

### Primary mouse tubular epithelial cell (TEC) isolation and culture

Mouse kidney TECs were isolated and cultured from WT C56BL/6, *Gpr43−/−* and *Gpr109A−/−* mice as described previously (Li et al., 2020). In brief, kidneys were perfused with saline then removed. Kidney cortices were dissected into 1 mm^3^ pieces and digested in HBSS containing 3 mg/mL of collagenase at 37°C for 25 min, followed by washing in DMEM/F12 medium (Invitrogen). The kidney digest was washed through a series of sieves (mesh diameters: 250, 150, 75 and 40 µm) then spun down at 300 g for 5 min. The cell pellet was re-suspended in defined K1 medium: DMEM/F12 supplemented with 25 ng/mL epidermal growth factor, 1 ng/mL PGE1, 5 x 10-11M triiodothyronine, 5 x 10-8M hydrocortisone (Sigma-Aldrich), ITSS media supplement, 1% penicillin/streptomycin, 25 mM HEPES, and 5% FCS (Invitrogen). Cell suspension was then seeded on cell culture Petri dishes and incubated at 37°C for 2–3 h to facilitate adherence of contaminating glomeruli. The non-adherent tubules were collected and cultured on collagen-coated Petri dishes (BD Biosciences) in K1 medium. Expression of the epithelial cell marker cytokeratin was verified by immunofluorescent staining with an anti-cytokeratin antibody (Sigma-Aldrich). Cells were >95% cytokeratin positive.

### Primary mouse podocyte isolation and culture

Kidneys were perfused with 10^7^ Dynabeads and the cortex was cut into small pieces (1–2 mm^3^) and digested in 2 mg/mL collagenase at 37°C for 30 min. The collagenase-digested tissue was passed through a 100 µm sieve and centrifuged at 200g. The pellet was resuspended and glomeruli-containing Dynabeads were gathered in a magnetic field. The glomeruli were pipetted onto a 40 µm nylon sieve to remove free Dynabeads and collected by washing through an inverted nylon sieve. Isolated glomeruli were seeded onto collagen-coated culture dishes (BD Biosciences) in DMEM/F-12 medium containing 5% fetal bovine serum supplemented with 0.5% insulin-transferrin-sodium selenite (ITSS), 100 U/mL penicillin and 100 mg/mL streptomycin (Invitrogen) and incubated at 37°C. The cultured cells were examined for the podocyte markers podocin and nephrin by immunofluorescent staining. Cells were >95% positive for these markers.

### High glucose stimulation of podocytes or TEC *in vitro*

Cultured podocytes or TECs at 80% confluence were rinsed and incubated with serum-free DMEM/F12 medium with 0.5% ITSS supplement for podocytes, or serum free K1 medium for TECs for 48 h. Cells were then exposed to 30 mM D-glucose (Invitrogen) or mannitol (5.5 mM glucose + 24.5 mM mannitol) in the presence and/or absence of acetate (25 mM), propionate (12 mM) or butyrate (3.2 mM) for 12 h (Andrade-Oliveira et al., 2015). After stimulation, cells were harvested for RNA extraction and assessment of mRNA expression by RT-PCR.

### Real-time RT-PCR

Total RNA was extracted from kidney tissue or cells using TRIzol reagent (Invitrogen, CA) according to the manufacturer’s instructions. cDNA was synthesized using oligo (dT)_16_ primers (Applied Biosystems) and the SuperScript III reverse transcriptase kit (Invitrogen) according to the manufacturer’s instructions. cDNA was amplified in universal Master Mix (Applied Biosystems) with gene-specific primers and probes, using the Roche Lightcycler 480 (Roche Applied Science). Specific TaqMan primers and probes for IL6, TNF-α, CCL2, CXCL10, TGF-β, fibronectin and GAPDH were used. Results were normalised to GAPDH expression.

### Statistical analysis

All results are expressed means ± SD or mean ± SEM. Data was analysed using student’s two-tailed t-tests, or one- or two-way analysis of variance (ANOVA) with posthoc Bonferroni’s correction (Graph Pad Software, San Diego, CA, United States of America) where appropriate. A *p*-value of < 0.05 was considered statistically significant.

## Results

### Wild-type, *Gpr43*−/− and *Gpr109A*−/− mice develop equivalent STZ-induced diabetes

**FIGURE 1:**
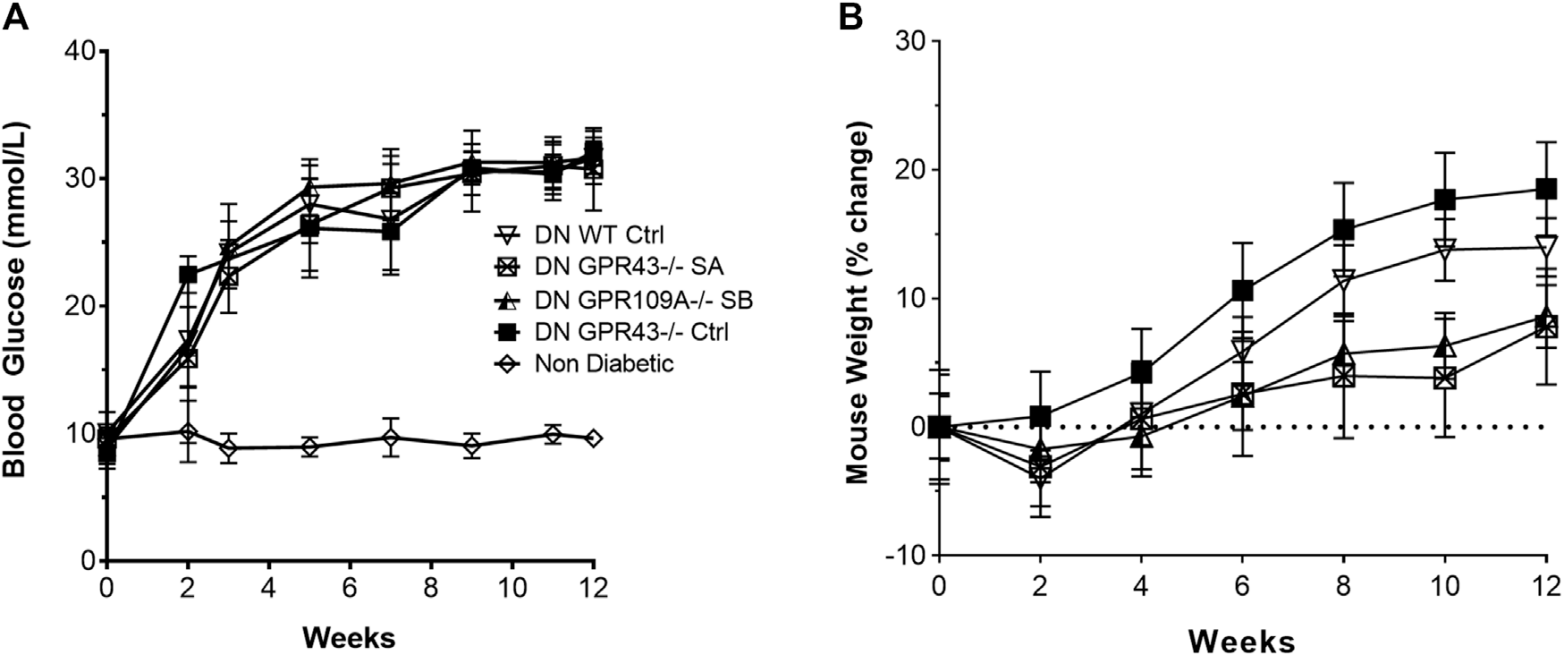
Wild-type, *Gpr43−/−* and *Gpr109A−/−* mice developed equivalent degrees of hyperglycaemia. STZ induced diabetes in WT, GPR43−/− and GPR109A−/− mice of similar severity, as indicated by blood glucose **(A)** and body weight **(B)** profiles over a 12-week period. *n* = 8–12 in diabetic groups. Age matched non-diabetic controls *n* = 5 mice per group. Data are shown as means ± SD.

WT, *Gpr43−/−* and *Gpr109A−/−* mice were equally susceptible to STZ -induced diabetes, displaying similar profiles in progression of hyperglycaemia (Figure 1A) and weight change (Figure 1B) over the 12-week experimental period. SCFA treatment did not alter the glucose profile relative to diabetic controls.

### Metabolite-sensing G-protein coupled receptors GPR43 and GPR109A are required for SCFA mediated protection against histological injury in diabetic nephropathy

In a previous study, we demonstrated that SCFAs protected against development of albuminuria and histological damage in STZ induced DN (4). SCFAs are known to exert some of their effects through metabolite sensing GPRs to elicit cellular responses. To determine whether these signaling pathways are involved in SCFA mediated renoprotection, we treated diabetic WT, *Gpr43−/−* and *Gpr109A−/−* mice with their primary ligands acetate and butyrate, respectively, supplemented in drinking water.

**FIGURE 2:**
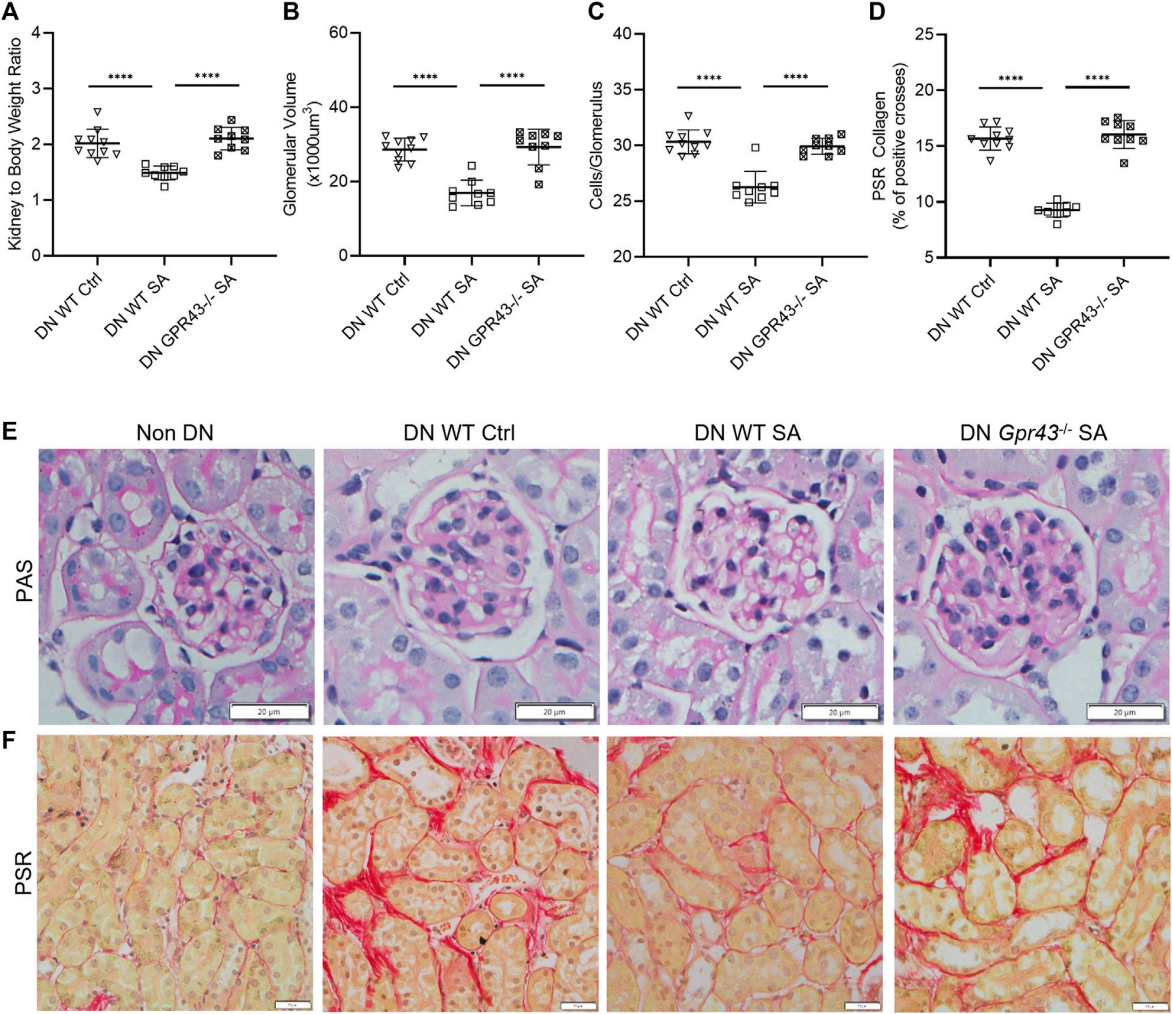
SCFA reduce glomerular and interstitial injury in DN through GPR43. Acetate was ineffective in ameliorating diabetic nephropathy in GPR43−/− diabetic mice, with no reduction in kidney to body weight ratio **(A)**, glomerular volume **(B)**, glomerular hyper-cellularity **(C)** or interstitial fibrosis **(D)** at 12 weeks compared to WT diabetic controls. **(E, F)** Representative sections of kidney from WT and GPR43−/− mice at 12 weeks (PAS and PSR stained, ×400 magnification).

**FIGURE 3:**
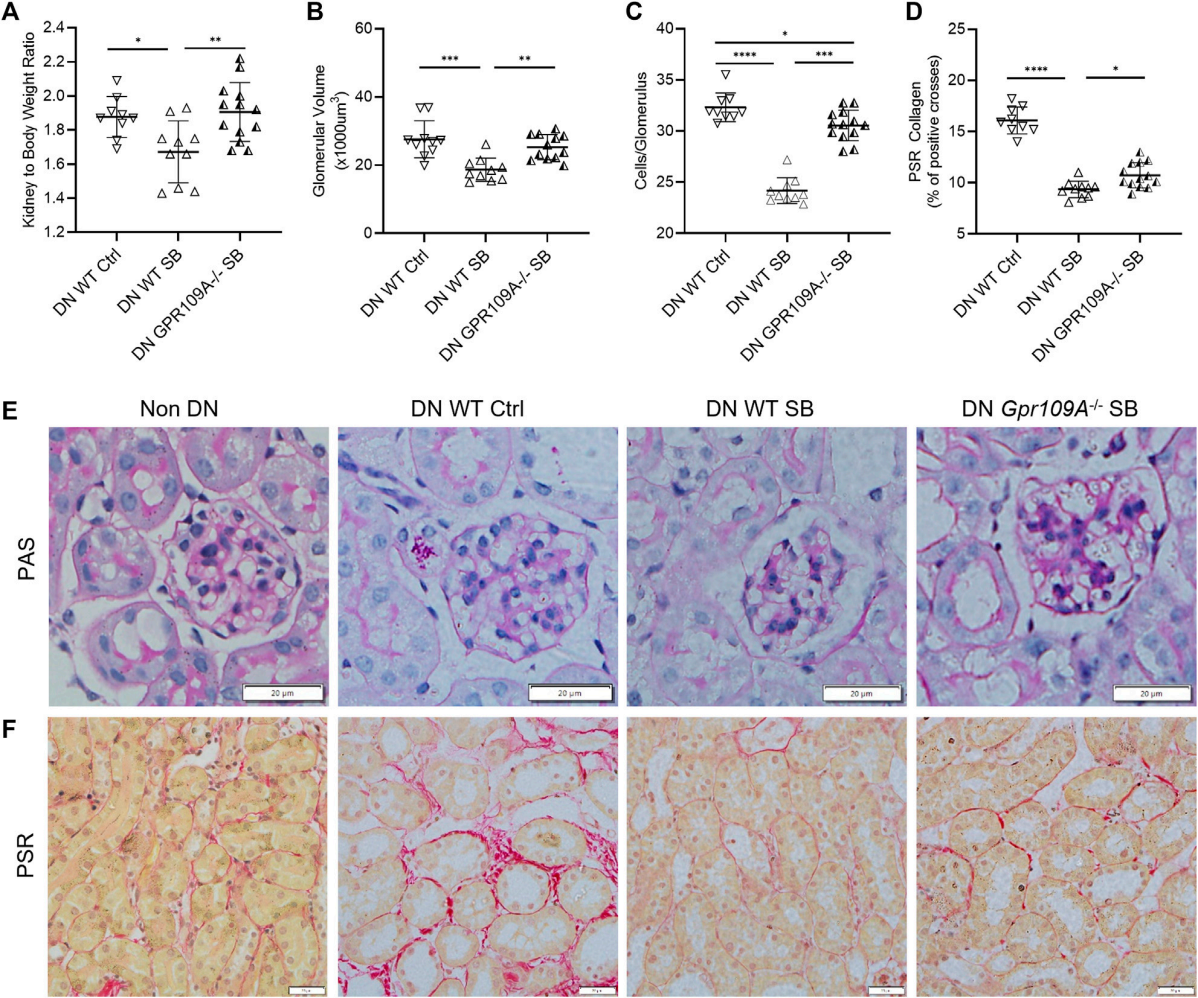
Butyrate reduces diabetic glomerular injury through GPR109A. Butyrate treatment in GPR109A−/− diabetic mice did not alter kidney to body weight ratio **(A)** but resulted in less glomerular hypertrophy **(B)** and glomerular hypercellularity **(C)** compared to WT diabetic controls. Increased interstitial collagen deposition was partially attenuated by butyrate treatment in GPR109A−/− diabetic mice **(D, F)**. **(E, F)** Representative sections of kidney from WT and GPR109A−/− mice at 12 weeks (PAS and PSR stained, ×400 magnification).

WT B6 mice treated with acetate were protected from development of DN with a lower kidney to body weight ratio, less glomerular hypertrophy and mesangial cellularity compared to WT diabetic controls (Figures 2A–C). However, acetate treatment in diabetic *Gpr43−/−* mice was ineffective, with similar histological injury severity to control WT diabetic mice, as evidenced by increased glomerular volume and mesangial cellularity (Figure 2E). *Gpr109A−/−* diabetic mice treated with butyrate exhibited similar kidney to bodyweight ratios to diabetic WT controls but developed less glomerular hypertrophy and mesangial cellularity compared to WT diabetic controls (Figures 3A–C, E). However there remained a significant difference between WT and *Gpr109A−/−* butyrate treated diabetic mice, indicating that butyrate provided partial protection against DN in the absence of *Gpr109A−/−*.

Quantification of interstitial fibrosis using Picro-Sirius Red staining revealed substantial interstitial collagen deposition in diabetic WT kidneys compared to non-diabetic WT kidneys. Diabetic *Gpr43−/−* kidneys revealed significant tubulointerstitial fibrosis with collagen deposition of similar severity compared with WT diabetic controls despite acetate treatment (Figures 2D, F). In contrast, diabetic *Gpr109A−/−* kidneys from butyrate treated mice revealed modest degrees of fibrosis which was reduced in severity compared with control WT diabetic kidneys (Figures 3D, F).

### Exposure to high glucose induces a pro-inflammatory response in WT, *Gpr43−/−* and *Gpr109A−/−* podocytes and TECs

**FIGURE 4:**
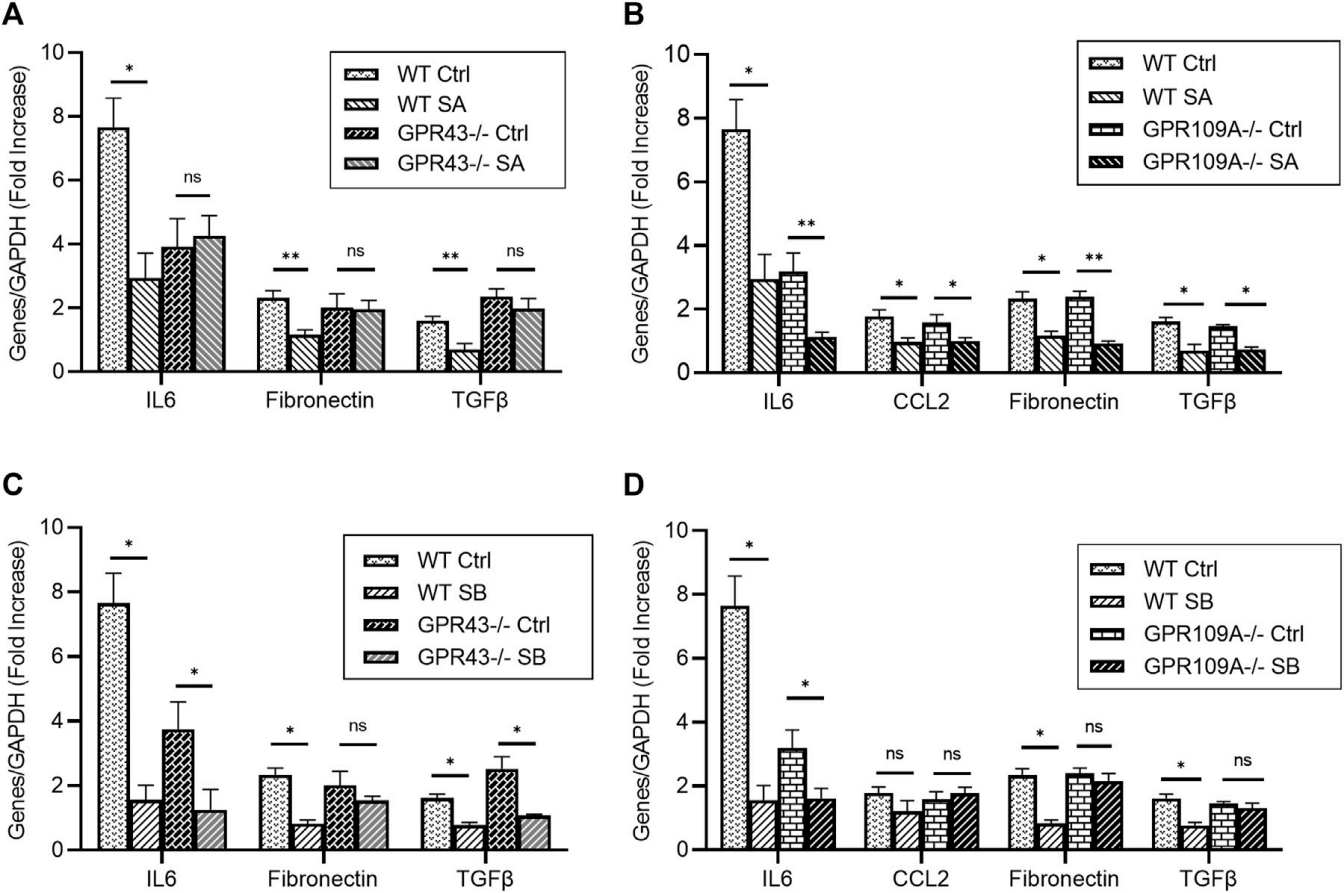
GPR43 and GPR109A are critical to inflammatory and fibrotic responses in glucose stimulated TECs. Primary TECs from WT, GPR109A−/− and GPR43−/− mice were exposed to high glucose or mannitol as an osmotic control in the presence of acetate or butyrate for 12 h **(A–D)** High glucose conditions induced upregulation of pro-inflammatory and pro-fibrotic genes 2-7-fold in TECs. Treatment with acetate and butyrate reduced expression of IL6, Fibronectin and TGFβ in WT TECs compared to osmotic controls. Acetate was unable to diminish expression of these genes in the absence of the G-protein coupled receptor GPR43 **(A)**, but was still effective in Gpr109A−/− TECs **(B)**. Butyrate mediated its effects on fibrosis related genes primarily through GPR109A, with no effect on Fibronectin and TGFβ mRNA expression in Gpr109A−/− TECs **(D)**. Data are shown as means ± SEM (*n* = 6); *p < 0.05, **p < 0.01.

**FIGURE 5:**
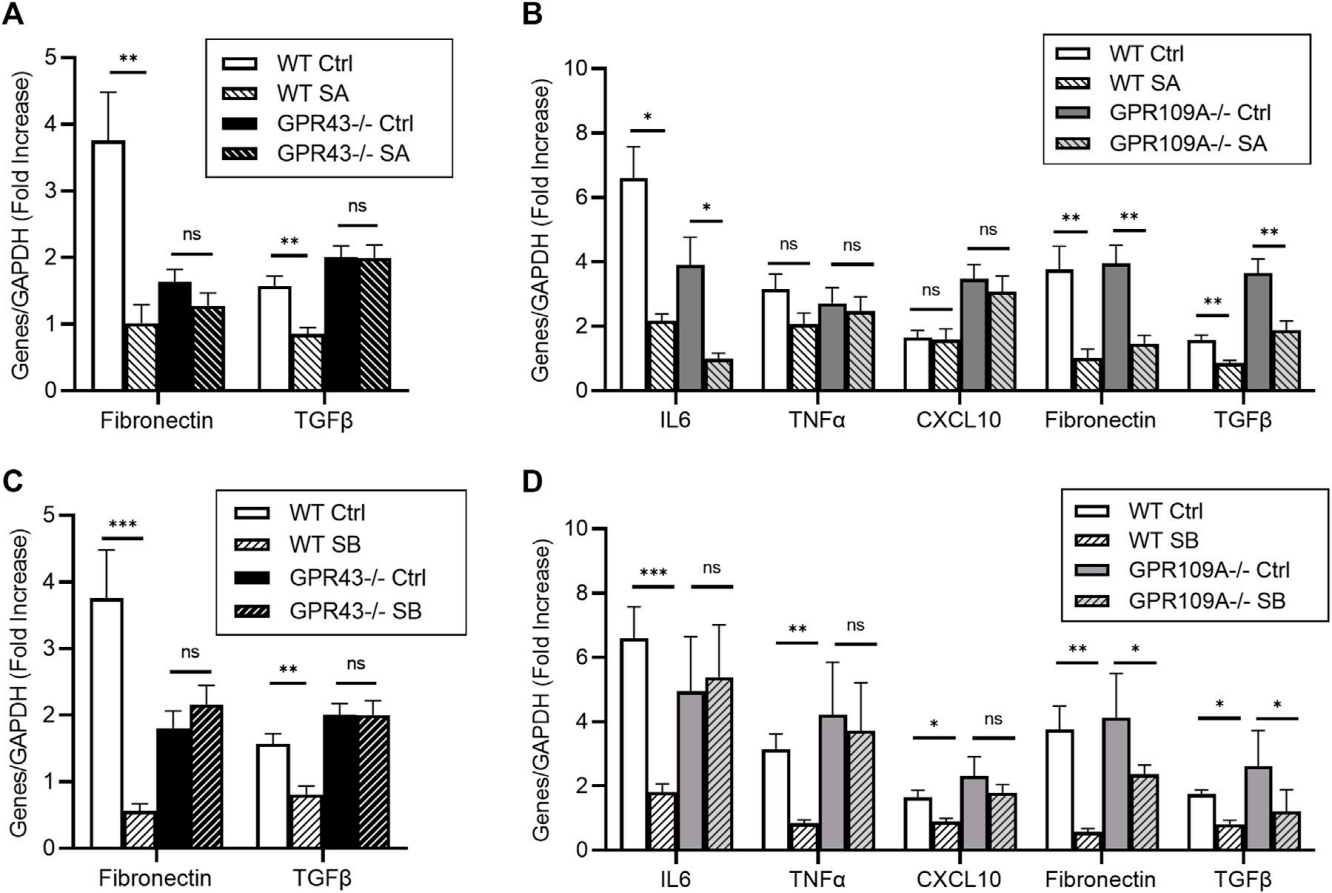
Acetate and butyrate influence pro-fibrotic genes in glucose stimulated podocytes via GPR43. Primary podocytes from WT, GPR109A−/− and GPR43−/− mice were cultured with high glucose or mannitol as an osmotic control in the presence or absence of acetate or butyrate for 12 h **(A–D)** Real-time PCR analysis demonstrated high glucose concentration induced upregulation of pro-inflammatory and pro-fibrotic genes IL6, TNFα, Fibronectin and TGFΒ, which was reduced by acetate and butyrate in WT podocytes. **(D)** Butyrate was unable to reduce IL6, TNFα and CXCL10 expression in GPR109A−/− podocytes, suggesting a GPR109A dependent mechanism. Absence of GPR109A did not impact acetate or butyrate’s effect on TGFβ and Fibronectin expression **(B, D)**. However, glucose induced upregulation of TGFβ and Fibronectin expression was not diminished by acetate and butyrate in podocytes derived from GPR43−/− mice **(A, C)**. Data are shown as means ± SEM (*n* = 6); *p < 0.05, **p < 0.01, ***p < 0.001.

To confirm whether SCFAs directly mediate protective responses through these signaling pathways in intrinsic kidney cells under hyperglycaemic conditions, primary tubular epithelial cells (TEC) and podocyte cultures from WT, *Gpr43*−/− and *Gpr109A*−/− kidneys were treated with acetate, butyrate, or propionate under normal or high glucose conditions. Exposure to high glucose increased mRNA expression of pro-inflammatory genes (IL6, TNFa) and pro-fibrotic genes (TGFβ, Fibronectin) 2-7-fold compared to osmotic controls (Figures 4, 5).

### Acetate and butyrate influence pro-inflammatory and pro-fibrotic gene expression in TECs exposed to high glucose through GPR43 and GPR109A

We have previously shown that SCFAs attenuated pro-inflammatory and pro-fibrotic responses in primary TECs exposed to high glucose. Acetate, butyrate, and propionate attenuated glucose induced upregulation of IL6, Fibronectin and TGFβ in WT TECs (Li et al., 2020). However, this effect was not seen in acetate treated *Gpr43−/−* TECs, with no reduction in IL6, Fibronectin or TGFβ expression compared to osmotic controls, suggesting a GPR43 dependent signaling mechanism (Figure 4A). Acetate treatment of *Gpr109A−/−* TECs resulted in diminished expression of IL6, CCL2, CXCL10, TGFβ and Fibronectin, consistent with the effect seen in WT TECs (Figure 4B).

Butyrate’s effects on pro-fibrotic responses appear GPR109A dependent, with butyrate unable to downregulate Fibronectin or TGFβ expression in *Gpr109A−/−* TECs (Figure 4D). In contrast, butyrate treatment in the absence of GPR109A or GPR43 had no effect pro-inflammatory gene expression. Butyrate attenuated IL6 expression in WT, *Gpr109A−/−* and *Gpr43−/−* derived TECs (Figures 4C, D), indicating butyrate facilitates its beneficial effects via both GPCRs and additional pathways.

### Acetate and butyrate attenuate pro-fibrotic podocyte responses in a GPR43 dependent manner

RT-PCR demonstrated glucose induced upregulation of pro-inflammatory and pro-fibrotic mRNA gene expression in primary podocytes (Figure 5). Whilst butyrate treatment attenuated expression of IL6, TNFα and CXCL10 expression in WT podocytes, it was ineffective *Gpr109A−/−* podocytes (Figure 5D). Absence of GPR109A did not alter expression of pro-inflammatory genes following treatment with acetate under high glucose conditions, as compared to WT controls (Figure 5B).

In contrast, whilst acetate and butyrate both suppressed expression of pro-fibrotic genes (Fibronectin, TGFβ) compared to osmotic controls in WT podocytes, this effect appeared independent of GPR109A, and was instead mediated through GPR43. Both SCFAs were able to suppress Fibronectin and TGFβ mRNA expression in *Gpr109A−/−* podocytes (Figures 5B, D) but were unable to alter TGFβ and Fibronectin expression in *Gpr43−/−* podocytes (Figures 5A, C).

## Discussion

Short-chain fatty acids are emerging as key players in gut-microbiota-host crosstalk and regulate immune and inflammatory responses in both health and disease (Sommer and Backhed, 2013; Lynch and Pedersen, 2016). We have previously demonstrated that fibre derived SCFAs limit renal inflammation and protect against nephropathy in an STZ-induced model of T1DM (Li et al., 2020). However, localization of the specific mechanistic sites by which SCFAs exert their reno-protective effects has not been well defined. In the present study, we found that GPRs GPR43 and GPR109A are critical to SCFA mediated protection against glomerular hypertrophy and interstitial fibrosis in DN, with mice lacking these receptors not afforded the same level of reno-protection. *In vitro* exposure of primary TECs and podocytes to high glucose stimulated inflammatory and pro-fibrotic gene expression, whilst treatment with the SCFAs acetate or butyrate suppressed these responses. Both acetate and butyrate were less effective in cells genetically deficient in GPR43 and GPR109A, supporting a role for tissue-specific GPR-dependent renal cellular signaling pathways in mediating protection.

SCFAs produced by fermentation of dietary fibre by the gut microbiota, are utilized locally as energy for colonocytes or absorbed via monocarboxylate transporters and travel via the portal circulation to mediate extra-intestinal effects (Cummings et al., 1987; Murase et al., 1995). Evidence supports their reno-protective potential in acute and chronic kidney diseases, and through influencing allo-immunity in kidney transplantation (Wu et al., 2020; Liu et al., 2021). In our study, oral supplementation of acetate, butyrate and propionate protected against the histological manifestations of DN *in vivo*, supporting their role as key mediators in gut microbiota-kidney crosstalk.

SCFAs are proposed to influence physiology through two predominant mechanisms; 1) signaling at local and distant cells through specific G-protein coupled receptors (Maslowski et al., 2009; Shapiro et al., 2014) or 2) by inhibiting histone-deacetylases (HDAC) with consequent impacts on epigenetic regulation of gene transcription, cell proliferation and apoptosis (Davie, 2003). The importance of GPRs in mediating SCFA’s effects on diabetic nephropathy was evident in our study, with protective effects attenuated in GPR43 and GPR109A deficient mice. GPR43 is known to be expressed in whole kidney tissue, cortical epithelial cells and arterioles (Kobayashi et al., 2017), whilst GPR109A expression has been demonstrated in kidney tissue and podocytes (Rajkumar et al., 2014; Felizardo et al., 2019). However, whether these reno-protective effects are due to local gut-derived, immune mediated or tissue specific signaling remained unclear.

To explore this further, we examined the effect of SCFAs on gene expression in primary TECs and podocytes exposed to high glucose. Consistent with the reno-protection seen *in vivo*, SCFAs acetate and butyrate attenuated pro-inflammatory and pro-fibrotic responses in glucose-stimulated TECs and podocytes. These effects were again dependent upon GPR43 and GPR109A. Other studies of renal cell lines *in vitro* have supported the importance of kidney specific GPR signaling. SCFA treatment of renal cortical epithelial cells reduced TNF-α induced MCP-1 expression, whilst both SCFAs and GPR43 agonists were able to inhibit cellular proliferation, production of ROS and expression of MCP-1 and IL-1β in glucose stimulated glomerular mesangial cells (Huang et al., 2017). Butyrate has been proposed to play a role mediating fibrosis, with reduced TGFβ1 generation in HK-2 epithelial cells exposed to butyrate (Matsumoto et al., 2006). We found butyrate was unable to attenuate fibrosis-related genes in *Gpr109A*−/− TECs *in vitro.* Worse fibrosis was also evident histologically in butyrate treated *Gpr109A*−/− diabetic mice as compared to butyrate treated diabetic WT controls, supporting a GPR109A-dependent effect.

Whilst our *in vitro* results suggest the presence of GPR-dependent renal signaling pathways, other gut derived, local or immune mediated mechanisms cannot be discounted, as studies *in vitro* may not truly represent conditions *in vivo*. We utilized SCFA doses in keeping with physiologic concentrations observed in the gut and systemically, and doses observed to alter expression of adhesion molecules and effector function in neutrophils *in vitro* (Cummings et al., 1987; Vinolo et al., 2009; Andrade-Oliveira et al., 2015). However, oral administration of SCFAs at higher doses have been found to induce ureteric inflammation and hydronephrosis (Park et al., 2016). Thus, whether SCFAs exert differing dose-dependent effects in DN remain to be explored.

In conclusion, SCFAs diminish inflammation in kidney tubular epithelial cells, podocytes and ameliorate progression of DN *in vivo* through GPR-dependent pathways. Our *in vitro* studies confirm that direct, GPR-dependent effects on intrinsic kidney cells are at least in part responsible for this protection. Modification of the gut microbiome and metabolome is a potential leverage point for clinical intervention. Thus, a greater understanding the mechanisms by which gut-derived metabolites such as SCFAs influence physiology and disease could have major implications for managing the global burden of diabetic kidney disease.

## Data Availability

The raw data supporting the conclusion of this article will be made available by the authors, without undue reservation.
